# Supplementary material for: WSX1 act as a tumor suppressor in hepatocellular carcinoma by downregulating neoplastic PD-L1 expression
Source: Nat Commun. 2021 Jun 9;12:3500. doi: 10.1038/s41467-021-23864-9 (PMC8190270; doi:10.1038/s41467-021-23864-9)
Supplement: Supplementary file 1 — Supplementary Information [file 41467_2021_23864_MOESM1_ESM.pdf]

Supplementary Information

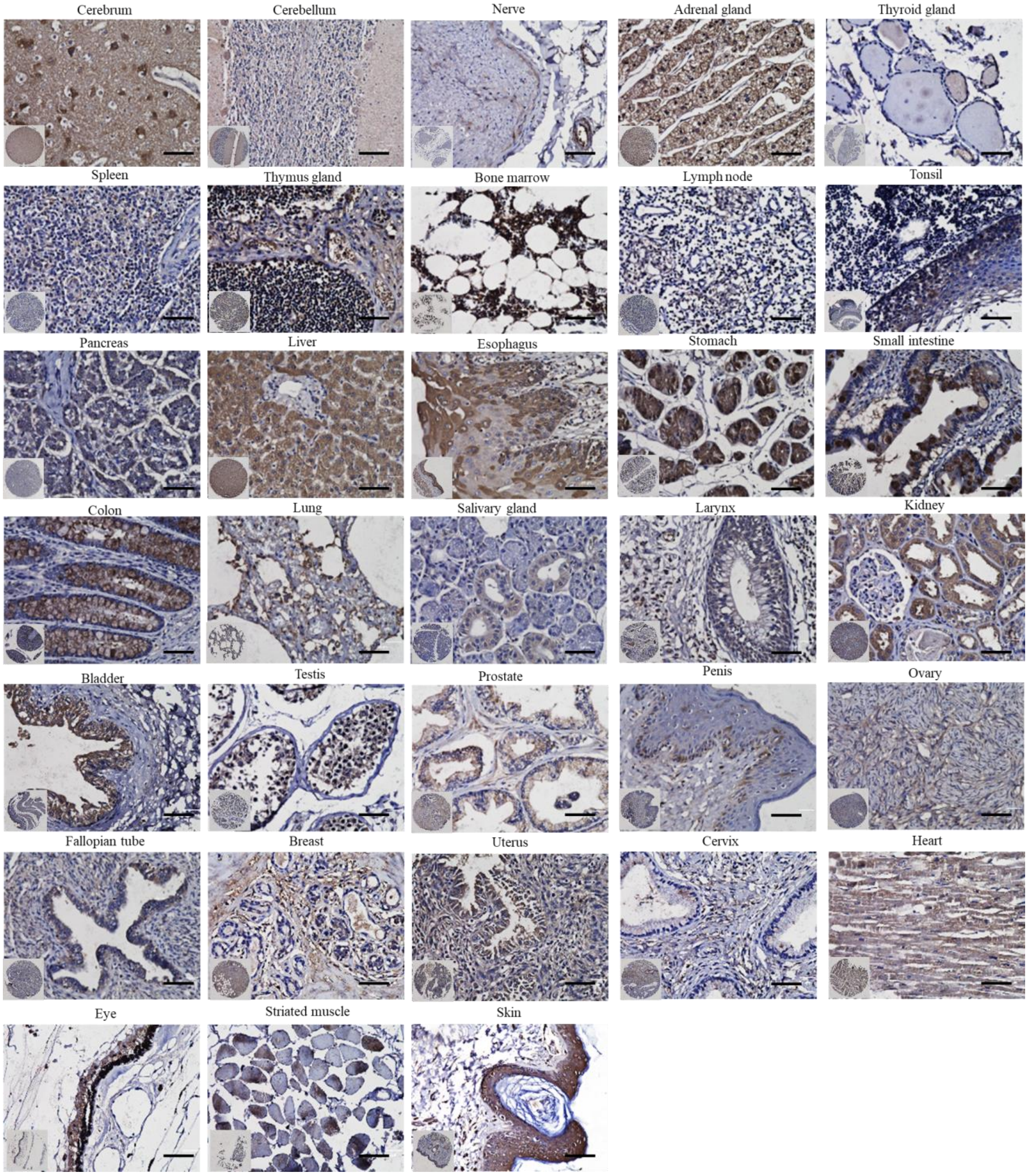

Supplementary Figure 1. Expression of WSX1 in multiple normal human organs. Immunohistochemical staining results of WSX1 in tissue microarray (FDA662a). FDA662a contains 33 types of normal human organs, with samples from each organ taken from 2 individuals. Scale bars, 50 μm.

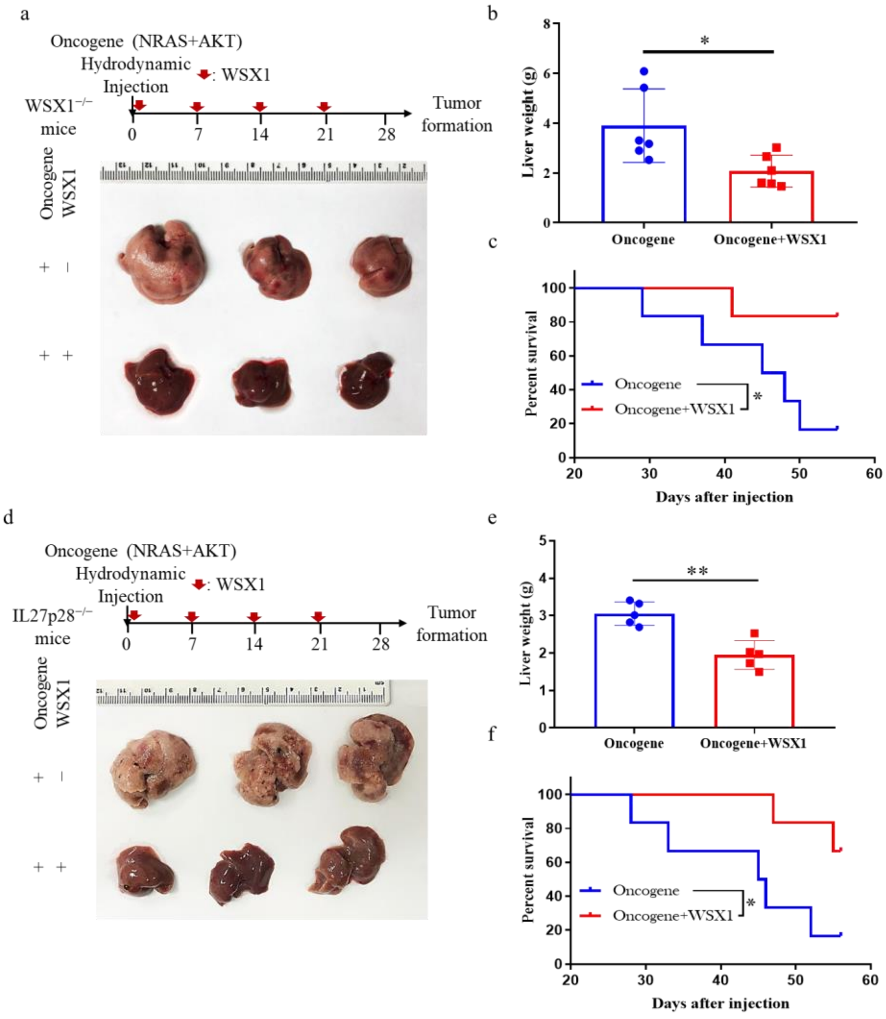

Supplementary Figure 2. The tumor-suppressive function of WSX1 is independent of IL-27. (a) (Top) Summary of *NRAS/AKT* oncogene–derived spontaneous HCC mouse model in WSX1$^{-/-}$ C57BL/6J mice. Arrowheads represent WSX1 injection once a week for 4-6 weeks. (Bottom) Representative images of entire livers in the oncogene and oncogene + WSX1 groups. **(b)** Effect of WSX1 on the oncogene-induced tumor formation in WSX1$^{-/-}$ C57BL/6J mice (n = 6, P = 0.0192). **(c)** Effect of WSX1 on the overall survival of WSX1$^{-/-}$ C57BL/6J mice (n = 6, HR = 0.1390, P = 0.0332). **(d)** (Top) Summary of HCC mouse model in IL27p28$^{-/-}$ C57BL/6J mice. (Bottom) Representative images of entire livers. **(e)** Influence of WSX1 on the oncogene-induced tumor formation in IL27p28$^{-/-}$ C57BL/6J mice (n = 5, P = 0.0011). **(f)** Difference of the overall survival between oncogene and oncogene+WSX1 group (n = 6, HR = 0.2074, P = 0.0326) in IL27p28$^{-/-}$ C57BL/6J mice. All data are representative of 2 independent experiments. Quantitative data are presented as mean ± SD and analyzed by the two-sided Student t test. The survival curves

were analyzed by the Kaplan-Meier method, and the log-rank test was used to compare overall survival between groups. * $P < 0.05$, ** $P < 0.01$. Source data are provided as a Source Data file.

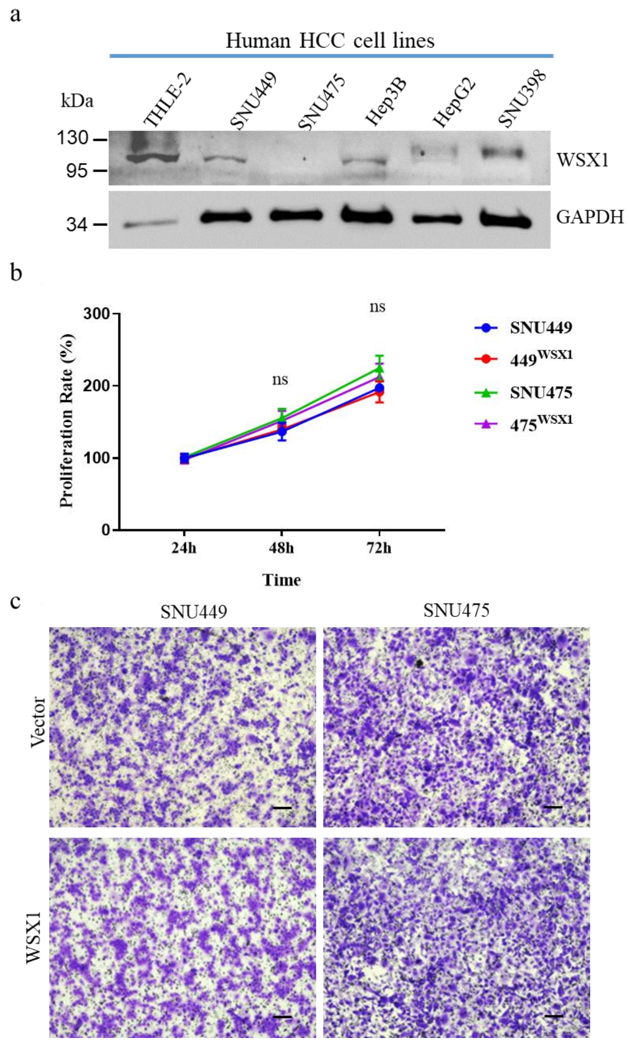

Supplementary Figure 3. WSX1 had no significant effect on proliferation and migration of HCC cells in vitro. (a) Expression of WSX1 in HCC and normal liver cell lines determined by Western blotting. THLE-2 cells were used as a relative normal control, which were derived from primary normal cells by infection with SV40 large T antigen. **(b)** CCK8 assay of the effect of WSX1 on HCC proliferation (n = 4 independent experiments). **(c)** Transwell assay of effect of WSX1 on HCC cell migration. Scale bars, 100 μm. All images shown are representative of 3 independent experiments. Quantitative data are presented as mean ± SD and were analyzed by two-sided Student *t* test; “ns” (not significant) represents $P > 0.05$. Source data are provided as a Source Data file.

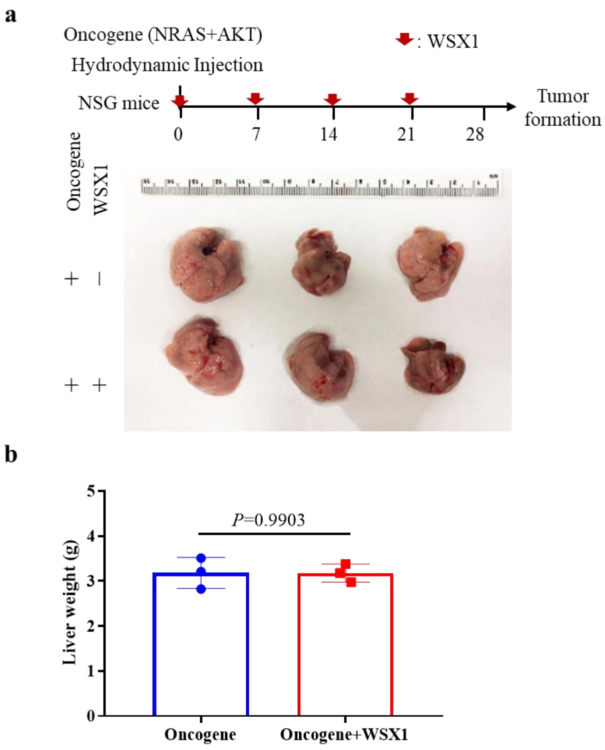

Supplementary Figure 4. WSX1 had no effect on oncogene-induced HCC development in NSG mice. (a) (Top) Summary of *NRAS/AKT* oncogene–derived spontaneous HCC mouse model in NSG mice. Arrowheads represent hydrodynamic injection of WSX1. (Bottom) Representative images of entire livers in the oncogene and oncogene + WSX1 groups. **(b)** Comparison of liver weight (n = 3). All data and images are representative of 2 independent experiments. Quantitative data are presented as mean ± SD and were analyzed by two-sided Student *t* test. Source data are provided as a Source Data file.

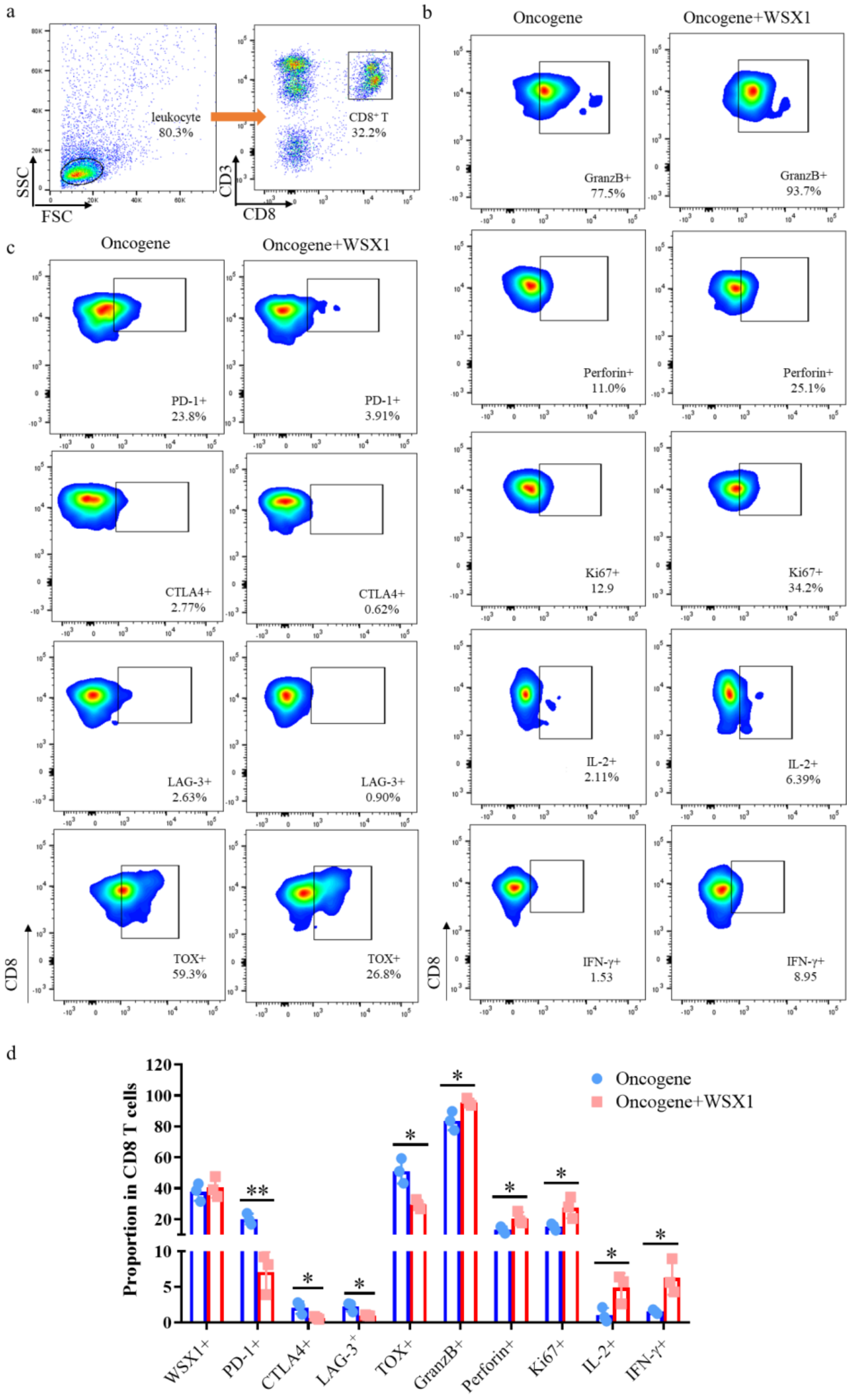

Supplementary Figure 5. WSX1 relieved CD8⁺ T-cell exhaustion in vivo. Intrahepatic immune cells were isolated from livers of spontaneous HCC mouse models. **(a)** The gating strategy for mouse intrahepatic CD8⁺ T-cells. **(b)** Expression of T-cell functional markers (granzyme B, perforin, Ki67, IL-2, and IFN-γ) in intrahepatic CD8⁺ T cells determined by flow cytometry. **(c)**

Expression of T-cell exhaustion markers (PD-1, CTLA4, LAG-3, and TOX) in intrahepatic CD8+ T cells analyzed by flow cytometry. **(d)** Statistical analysis of expression of T-cell functional markers, T-cell exhaustion markers, and WSX1 in intrahepatic CD8+ T-cells (n = 5). WSX1 significantly reduced the proportion of PD-1+ (*P* = 0.0074), CTLA4+ (*P* = 0.0047), LAG-3+ (*P* = 0.0295), and TOX+ CD8+ T-cells (*P* = 0.0116), while increased the percentage of GranzB+ (*P* = 0.0344), perforin+ (*P* = 0.0439), Ki67+ (*P* = 0.0418), IL-2+ (*P* = 0.0405), and IFN-γ+ CD8+ T-cells (*P* = 0.0268). All data are representative of 3 independent experiments. Quantitative data are presented as mean ± SD and were analyzed by two-sided Student *t* test. *$P < 0.05$, **$P < 0.01$. GranzB: granzyme B. Source data are provided as a Source Data file.

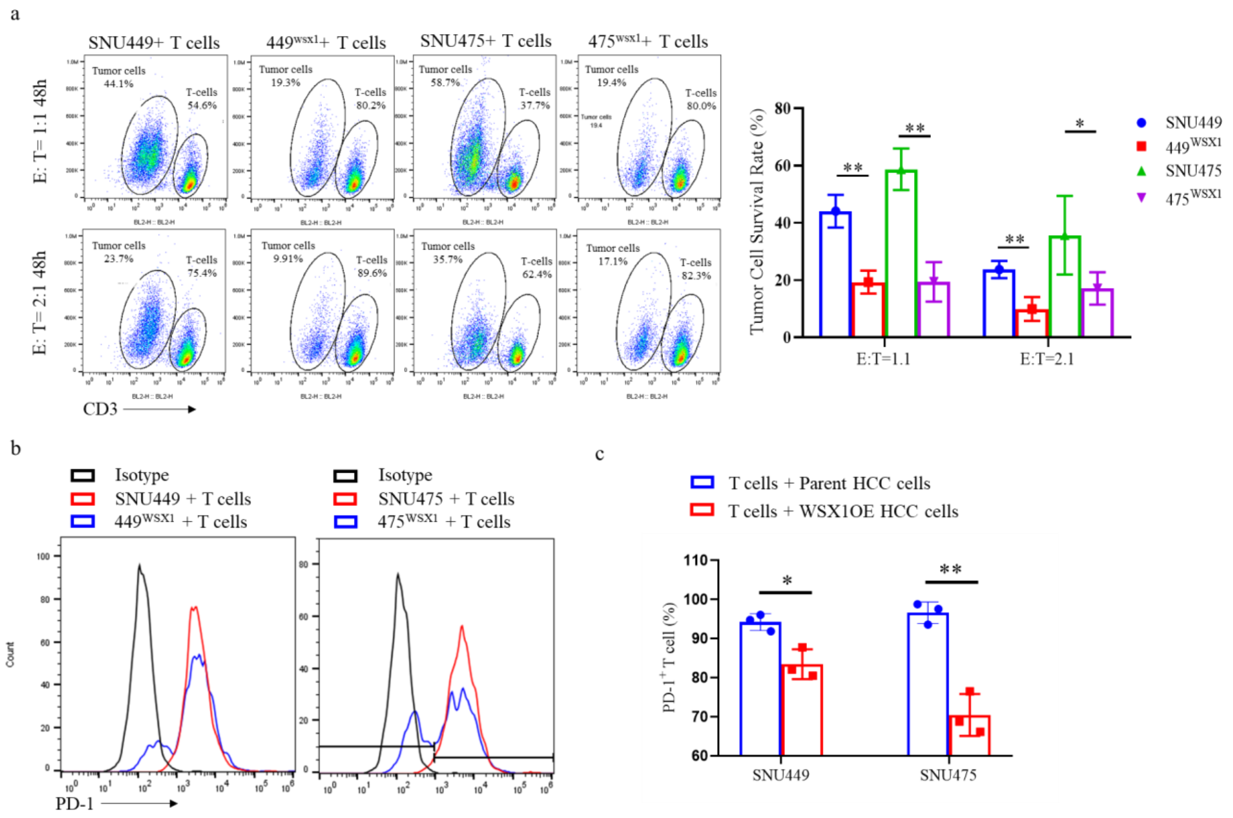

Supplementary Figure 6. WSX1 overexpression sensitized tumor cells to T cell–mediated killing and reduced PD-1 expression in T cells. (a) Effect of WSX1 on T-cell antitumor activity determined by T cell–mediated tumor cell killing assay. Activated human T-cells (E) were mixed with the indicated HCC cells (T) at different ratios (E:T = 1:1 or 2:1) and co-cultured in 12-well plates for 48 hours. After co-culture, a SYTOX™ AADvanced™ Dead Cell Stain Kit was used to exclude dead cells, and then anti-human CD3-specific antibodies were used to distinguish live T-cells from live HCC cells. The left ovals represent tumor cells (CD3−), and the right ones represent T-cells (CD3+). WSX1 overexpression significantly decreased HCC cell survival rates in both SNU449 (n = 3 independent experiments; E:T = 1:1, *P* = 0.0029; E:T = 2:1, *P* = 0.0108) and SNU475 cells (E:T = 1:1, *P* = 0.0022; E:T = 2:1, *P* = 0.0038). **(b)** Flow cytometric analysis of the

proportion of PD-1$^+$ T cells. Activated human T-cells were co-cultured with HCC cells (E:T = 1:1) for 48 hours and then analyzed by flow cytometry. **(c)** WSX1 overexpression in HCC cells reduced PD-1 expression of T cells in both SNU449 (n = 3 independent experiments, P = 0.0131) and SNU475 cells (P = 0.0017). All data and images are representative of 3 independent experiments. Quantitative data are presented as mean ± SD and were analyzed by two-sided Student t test. $*P < 0.05$, $**P < 0.01$. E, human effector T-cells; T, HCC cells. The gating strategy for sorting T-cells and HCC cells in the co-culture system is shown in supplementary Figure 8c. Source data are provided as a Source Data file.

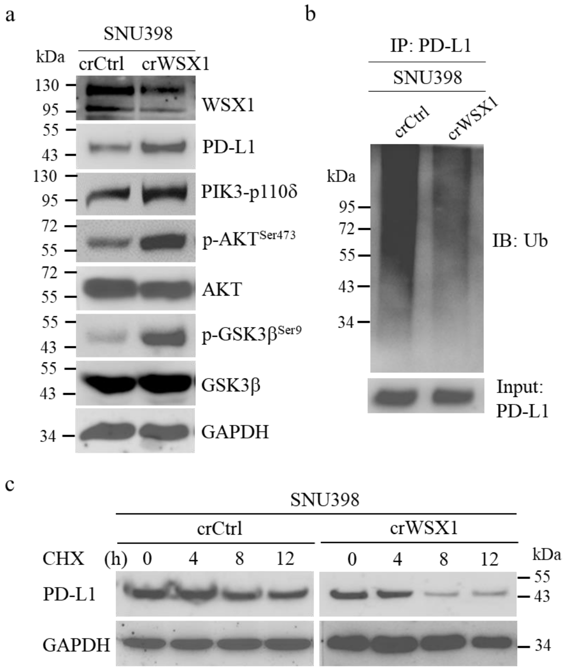

Supplementary Figure 7. Knockdown of WSX1 stabilized PD-L1 protein and upregulated PI3Kδ/AKT/GSK3β signaling pathway. (a) Effect of WSX1 knockdown on expression of PD-L1, PI3K-p100δ, p-AKT^{ser473}, AKT, p-GSK3β^{ser9}, and GSK3β in SNU398 cells. **(b)** Effect of WSX1 knockdown on PD-L1 ubiquitination. **(c)** Impact of WSX1 knockdown on PD-L1 protein degradation. Cells were treated with 25 mmol/L CHX for 0, 4, 8, and 12 h, and cell lysates were collected separately and analyzed for PD-L1 protein levels. CHX, cycloheximide. All images are representative of 3 independent experiments. h, hour. Source data are provided as a Source Data file.

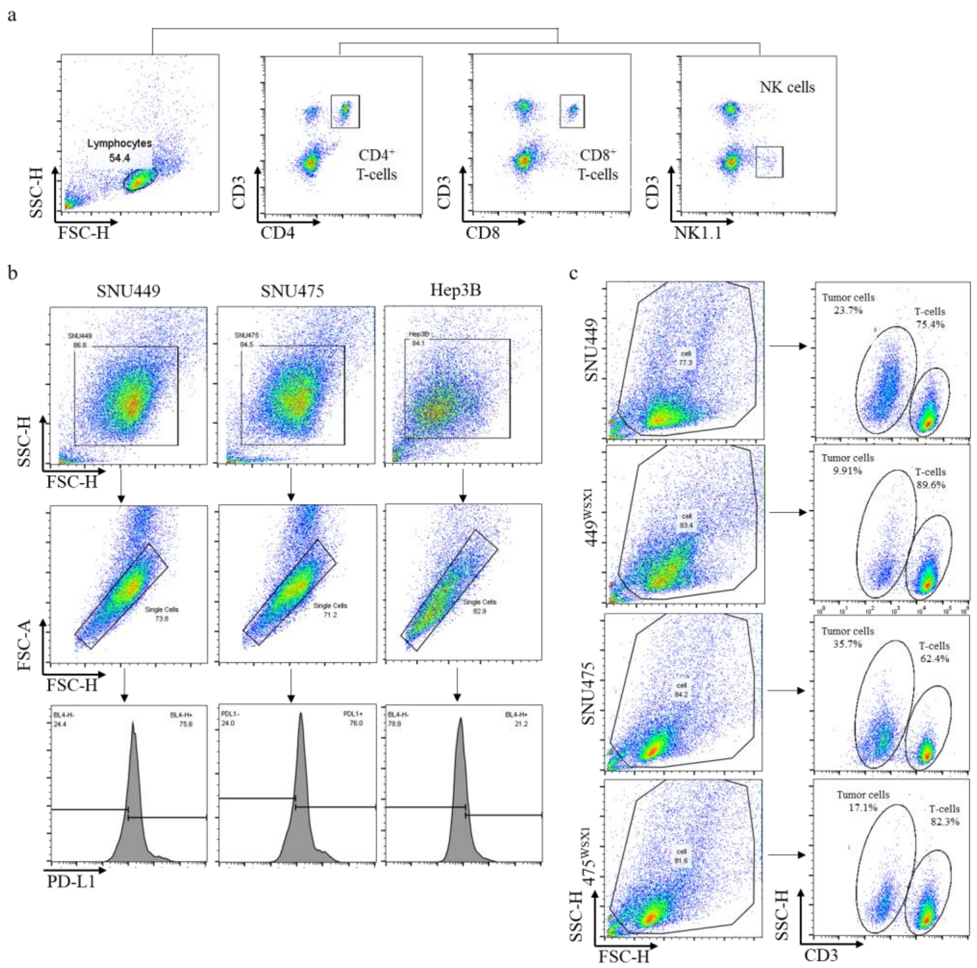

Supplementary Figure 8. Gating strategies used for cell sorting. (a) Gating strategy for sorting CD4^{+} T-cells, CD8^{+} T-cells and NK cells in Figure 4a. **(b)** Gating strategy for sorting PD-L1^{+} HCC cells in SNU449, SNU475 and SNU398 cells in Figure 5-7. **(c)** Gating strategy for sorting tumor cells and T-cells in Supplementary Figure 6.

Supplementary Table 1. Dilution of antibodies.

Antibodies	Label	Vendor	Catlogue No.	Dilutions
PD-L1	/	Proteintech	17952-1-AP	IP：1μg/g protein
PD-L1	/	Proteintech	66248-1-Ig	1：1000
pan-AKT	/	Cell Signaling Technology	4685	1：1000
p-AKT^{S473}	/	Abcam	ab81283	1：1000
GSK3β	/	Cell Signaling Technology	12456	1：1000
p-GSK3β^{Ser9}	/	Cell Signaling Technology	5558	1：1000
WSX1	/	Thermo Fisher	PA5-96963	1：1000
β-catenin	/	Cell Signaling Technology	8480	1：1000
p-β-catenin$^{Ser33/37/Thr41}$	/	Cell Signaling Technology	9561	1：1000
PTEN	/	Cell Signaling Technology	9188	1：1000
p-TSC2^{Thr1462}	/	Cell Signaling Technology	3617	1：1000
TSC2	/	Cell Signaling Technology	4308	1：1000
PI3K-p85	/	Cell Signaling Technology	4292	1：1000
PI3K-p110α	/	Cell Signaling Technology	4255	1：1000
PI3K-p110δ	/	Cell Signaling Technology	34050	1：1000
FLAG	/	Cell Signaling Technology	2368	1：1000
Ub	/	Abcam	sc-8017	1：400
GAPDH	HRP	Proteintech	HRP-6000	1：10000
CD8a	V450	Tonbo	75-0081	0.25 μg/10^6cells
CD4	FITC	BioLegend	100405	0.25 μg/10^6cells
NK1.1	eFluor 450	ebioscience	48-5941-82	1 μg/10^6cells
PD-1	PE/Cy7	BioLegend	109109	1 μg/10^6cells
CTLA-4	PE	BioLegend	106306	1 μg/10^6cells
LAG-3	PE/Cy7	BioLegend	125225	0.5 μg/10^6cells
Tim3	PE	BioLegend	134009	0.5 μg/10^6cells
granzyme B	PE	ebioscience	12-8898-80	0.2 μg/10^6cells
Ki67	PE	BioLegend	652404	0.5 μg/10^6cells
perforin	PE	ebioscience	12-9392-82	0.5 μg/10^6cells
CD3ε	PerCP/Cyanine5.5	BioLegend	100328	0.25 μg/10^6cells
TOX	APC	Miltenyi Biotec	130-118-335	0.5 μg/10^6cells
IFN-γ	APC	ebioscience	17-7311-82	0.125 μg/10^6cells
IL-2	PE	BioLegend	503808	0.5 μg/10^6cells
CD3	PE	BioLegend	300308	5μl/10^6cells
PD-1	PE/Cy7	BioLegend	367414	5μl/10^6cells
WSX1	PE	R&D	FAB14791P	10μl/10^6cells
CD8α	/	BioXCell	clone 2.43	200 μg per mouse
CD4	/	BioXCell	clone GK1.5	200 μg per mouse
NK1.1	/	BioXCell	clone PK136	200 μg per mouse

Supplementary Table 2. Antibodies Used for Time-of-Flight Mass Cytometry Analysis

Marker	Clone	Label
Immune cell panel		
CD45	30-F11	89Y
CD4	RM4-5	115In
CD11b	M1/70	139La
Gr-1	RB6-8C5	141Pr
CD11c	N418	142Nd
GITR	DTA1	143Nd
CD68	FA-11	145Nd
CD8a	53-6.7	146Nd
LAG-3	C9B7W	147Sm
CD27	LG.3A10	148Nd
OX40	OX-86	149Sm
CD25	3C7	150Nd
CD123	5B11	151Eu
CD3e	145-2C11	152Sm
PD-L1	10F.9G2	153Eu
CCR7	4B12	155Gd
CD69	H1.2F3	156Gd
Foxp3	FJK-16s	158Gd
NKp46	29A1.4	159Tb
TCRgd	eBioGL3	160Gd
CXCR3	CXCR3-173	161Dy
CTLA-4	9H10	163Dy
CD62L	MEL-14	164Dy
4-1BB	17B5	165Ho
CD44	IM7	166Er
WSX1	263503	167Er
CD206	C068C2	169Tm
CD127	A7R34	170Er
PD-1	29F.1A12	171Yb
Ki67	B56	172Yb
Granzyme B	GB11	173Yb
MHC-II	M5/114.15.2	174Yb
CD28	37.51	175Lu
B220	RA3-6B2	176Yb
Tumor cell panel		
p-AKT	M89-61	144Nd
PD-L1	10F.9G2	153Eu
CD44	IM7	166Er
WSX1	263503	167Er
Ki67	B56	172Yb
