# Supplementary material for: WSX1 act as a tumor suppressor in hepatocellular carcinoma by downregulating neoplastic PD-L1 expression
Source: Nat Commun. 2021 Jun 9;12:3500. doi: 10.1038/s41467-021-23864-9 (PMC8190270; doi:10.1038/s41467-021-23864-9)
Supplement: Supplementary file 3 — Reporting Summary [file 41467_2021_23864_MOESM3_ESM.pdf]

nature research

Corresponding author(s): DBPR

Last updated by author(s): May 16, 2021

Reporting Summary

Nature Research wishes to improve the reproducibility of the work that we publish. This form provides structure for consistency and transparency in reporting. For further information on Nature Research policies, see our Editorial Policies and the Editorial Policy Checklist.

Statistics

For all statistical analyses, confirm that the following items are present in the figure legend, table legend, main text, or Methods section.

n/a	Confirmed	
☐	☒	The exact sample size (n) for each experimental group/condition, given as a discrete number and unit of measurement
☐	☒	A statement on whether measurements were taken from distinct samples or whether the same sample was measured repeatedly
☐	☒	The statistical test(s) used AND whether they are one- or two-sided<br>*Only common tests should be described solely by name; describe more complex techniques in the Methods section.*
☐	☒	A description of all covariates tested
☐	☒	A description of any assumptions or corrections, such as tests of normality and adjustment for multiple comparisons
☐	☒	A full description of the statistical parameters including central tendency (e.g. means) or other basic estimates (e.g. regression coefficient) AND variation (e.g. standard deviation) or associated estimates of uncertainty (e.g. confidence intervals)
☐	☒	For null hypothesis testing, the test statistic (e.g. F, t, r) with confidence intervals, effect sizes, degrees of freedom and P value noted<br>*Give P values as exact values whenever suitable.*
☒	☐	For Bayesian analysis, information on the choice of priors and Markov chain Monte Carlo settings
☐	☒	For hierarchical and complex designs, identification of the appropriate level for tests and full reporting of outcomes
☐	☒	Estimates of effect sizes (e.g. Cohen's d, Pearson's r), indicating how they were calculated

Our web collection on statistics for biologists contains articles on many of the points above.

Software and code

Policy information about availability of computer code

Data collection	No software was used
Data analysis	GraphPad Prism 7 software (GraphPad Software, La Jolla, CA) and R package software (R Development Core Team, Version 3.5.3). Cytokit (https://github.com/hammerlab/cytokit).

For manuscripts utilizing custom algorithms or software that are central to the research but not yet described in published literature, software must be made available to editors and reviewers. We strongly encourage code deposition in a community repository (e.g. GitHub). See the Nature Research guidelines for submitting code & software for further information.

Data

Policy information about availability of data

All manuscripts must include a data availability statement. This statement should provide the following information, where applicable:

- Accession codes, unique identifiers, or web links for publicly available datasets
- A list of figures that have associated raw data
- A description of any restrictions on data availability

The authors declare that all data supporting the findings of this study are available within the paper and its Supplementary Information files or from the corresponding author upon reasonable request.

Field-specific reporting

Please select the one below that is the best fit for your research. If you are not sure, read the appropriate sections before making your selection.

- [x] Life sciences
- [ ] Behavioural & social sciences
- [ ] Ecological, evolutionary & environmental sciences

For a reference copy of the document with all sections, see nature.com/documents/nr-reporting-summary-flat.pdf

Life sciences study design

All studies must disclose on these points even when the disclosure is negative.

Sample size

For the the clinical data analysis, animal and cell line studies, sample sizes were decided based on the similar studies reported in the previous literature. Given 3-8 repeated or mice per group, we have 80% power to detect a difference in means between two groups in the range of 3.0-1.4*sd at a significance level of 0.05 using two-sample t-test. For the survival analysis, we used all the data from the cohort (90 patients each group), if the true hazard ratio (relative risk) of control subjects relative to experimental subjects is 0.4, we will be able to reject the null hypothesis that the experimental and control survival curves are equal with probability (power) .999. The Type I error probability associated with this test of this null hypothesis is 0.05.

Data exclusions

We removed outliers if the data were not met the assumption for homogeneity of variances.

Replication

Three different cell lines were used for testing reproducibility of our findings. All data shown in our study are representative of at least 3 independent experiments.

Randomization

Same age and gender of mice were randomly distributed into different groups.

Blinding

The data analysts were blinded to injected plasmids in spontaneous mouse models

Reporting for specific materials, systems and methods

We require information from authors about some types of materials, experimental systems and methods used in many studies. Here, indicate whether each material, system or method listed is relevant to your study. If you are not sure if a list item applies to your research, read the appropriate section before selecting a response.

Materials & experimental systems

n/a	Involved in the study	
	x	Antibodies
	x	Eukaryotic cell lines
x		Palaeontology and archaeology
	x	Animals and other organisms
x		Human research participants
x		Clinical data
x		Dual use research of concern

Methods

n/a	Involved in the study	
x		ChIP-seq
	x	Flow cytometry
x		MRI-based neuroimaging

Antibodies

Antibodies used

The following antibodies were used: primary antibody against PD-L1 (Proteintech 17952 and 66248), pan-AKT (Cell Signaling Technology 4685), phosphor-AKTS473 (Abcam ab81283, Cell Signaling Technology 9271), phosphor-AKTT308 (Cell Signaling Technology 9275), GSK3β (Cell Signaling Technology 12456), phosphor-GSK3βSer9 (Cell Signaling Technology 5558), WSX1 (Thermo Fisher PA5-96963), β-catenin (Cell Signaling Technology 8480), phosphor-β-cateninSer33/37/Thr41 (Cell Signaling Technology 9561), PTEN (Cell Signaling Technology 9188), phospho-TSC2Thr1462 (Cell Signaling Technology 3617), TSC2 (Cell Signaling Technology 4308), PI3K-p85 (Cell Signaling Technology 4292), PI3K-p110α (Cell Signaling Technology 4255), PI3K-p110δ (Cell Signaling Technology 34050), FLAG (Cell Signaling Technology 2368), Ub (Santa Cruz Biotechnology sc-8017), and WSX1 (clone 237, Monoclonal Antibodies Core Facility at MD Anderson Cancer Center)1. PerCP/Cyanine5.5 anti-mouse CD3 (BioLegend 100328), V450 anti-mouse CD8a (Tonbo 75-0081), FITC anti-mouse CD4 (BioLegend 100405), V450 anti-mouse NK1.1 (BD 560524), PE/Cy7 anti-mouse PD-1 (BioLegend 109109), PE anti-mouse CTLA-4 (BioLegend 106306), PE/Cy7 anti-mouse LAG-3 (BioLegend 125225), PE anti-mouse Tim3 (BioLegend 134009), PE anti-mouse granzyme B (ebioscience 12-8898-80), PE anti-mouse Ki67 (BioLegend 652404), PE anti-mouse perforin (ebioscience 12-9392-82), PE anti-human CD3 (BioLegend 300308), PE/Cy7 anti-human PD-1 (BioLegend 367414), PE anti-human WSX1 (R&D FAB14791P), and HRP anti-human/mouse GAPDH (Proteintech HRP-6000). Anti-mouse CD8α (clone 2.43), anti-mouse CD4 (clone GK1.5), and anti-mouse NK1.1 (clone PK136) for immune cell depletion were purchased from BioXCell. Mouse anti-human/mouse WSX1 was provided by Dr. Fred de Sauvage (Genentech).

Validation

Validations were performed by the providers.

Eukaryotic cell lines

Policy information about cell lines

Cell line source(s)	Human HCC cell lines SNU398, SNU449, SNU475, Hep3B, HepG2 and normal liver cell line THLE-2 were purchased from ATCC
Authentication	Cell lines were authenticated by the providers.
Mycoplasma contamination	Cells were treated with mycoplasma removal reagents before using.
Commonly misidentified lines (See ICLAC register)	N/A

Animals and other organisms

Policy information about studies involving animals; ARRIVE guidelines recommended for reporting animal research

Laboratory animals	C57BL/6J, FVB/NJ, and NOD/SCID mice were purchased from the Jackson Laboratory. WSX1-/- mice in C57BL/6J background were previously donated by Dr. Frederic de Sauvage (Genentech). IL-27p28-/- mice were generated as previously described by Jun Yan. All mice were aged 6 to 8 weeks when the experimental procedures began. Both gender were used in experiments.
Wild animals	N/A
Field-collected samples	N/A
Ethics oversight	All mice were maintained and treated in accordance with guidelines approved by the Institutional Animal Care and Use Committee at MD Anderson.

Note that full information on the approval of the study protocol must also be provided in the manuscript.

Flow Cytometry

Plots

Confirm that:

- [x] The axis labels state the marker and fluorochrome used (e.g. CD4-FITC).
- [x] The axis scales are clearly visible. Include numbers along axes only for bottom left plot of group (a 'group' is an analysis of identical markers).
- [x] All plots are contour plots with outliers or pseudocolor plots.
- [x] A numerical value for number of cells or percentage (with statistics) is provided.

Methodology

Sample preparation	Cells were collected and stained with the indicated fluorescence-conjugated primary antibodies for 30 minutes at 4°C in the dark. After being washed with PBS.
Instrument	Labeled cells were analyzed on an Attune acoustic focusing cytometer (Applied Biosystems)
Software	BD FACSDiva 8.0.1 and FlowJo V10
Cell population abundance	WSX1-stable transfectants in SNU449 and SNU475 cells enriched by FACSAria Cell Sorter (BD Biosciences) were over 90% as detected by FCS. The purities of CD3+T cells isolated from human peripheral blood mononuclear cells were over 95% determined by FCS.
Gating strategy	The Fluorescence Minus One Control, or FMO control were used to define the boundaries between positive and negative cell populations in mutiple fluorochromes panel.

- [x] Tick this box to confirm that a figure exemplifying the gating strategy is provided in the Supplementary Information.
